# Integrating video tracking and GPS to quantify accelerations and decelerations in elite soccer

**DOI:** 10.1038/s41598-021-97903-2

**Published:** 2021-09-17

**Authors:** Eduard Pons, Tomás García-Calvo, Francesc Cos, Ricardo Resta, Hugo Blanco, Roberto López del Campo, Jesús Díaz-García, Juan José Pulido-González

**Affiliations:** 1 Sports Performance Area, FC Barcelona, Barcelona, Spain; 2 Faculty of Sport Sciences, University of Extremadura, Avda. de la Universidad, S/N, 10003 Caceres, Spain; 3 Manchester City Football Club, Manchester, UK; 4 National Institute of Physical Education of Catalonia (INEFC), University of Barcelona, Barcelona, Spain; 5 Sports Research Area of LaLiga, Madrid, Spain; 6 Faculty of Human Kinetics, University of Lisbon, Lisbon, Portugal

**Keywords:** Biological techniques, Computational biology and bioinformatics

## Abstract

The aim of this study was to analyze the degree of agreement comparing number and distance covered in different acceleration and deceleration sections registered by a video tracking system (MEDIACOACH) and a GPS device (WIMU PRO) during official competition. Data from a Spanish professional club were registered over the course of a season. First, the descriptive statistics presented more bursts of accelerations and decelerations in WIMU PRO than in MEDIACOACH, whereas the distances covered recorded by both systems were similar. Second, negative relationships were found (i.e., negative bias) comparing WIMU PRO to MEDIACOACH in the number of accelerations and decelerations between 0/1 m/s^2^ and ½ m/s^2^ (*p* < 0.05), and in the distances covered in accelerations and decelerations (*p* < 0.05) between 0/1 m/s^2^ and in accelerations and decelerations registered between 2/3 m/s^2^ and more than 3 m/s^2^. Moreover, the differences in means (i.e., standardized mean bias) across the two devices were *trivial* (> 0.19) and *small* (0.2–0.59) for most variables. The standardized typical errors in the estimate (TEE) were *moderate* (0.3–0.59) and *small* to *moderate* (0.1–0.29 to 0.3–0.59), respectively. Also, the Intra class Correlation Coefficients (ICCs) for agreement and consistency between systems showed *good* and *excellent* values (> 0.90). The magnitude of change in means (%) between systems, defined as the percentage change between the numbers or values, was below 14% and 7% for number and distances covered, respectively. All scores in the smallest worthwhile change were lower than 9% and in the coefficients of variation were lower than 95% and 15%, respectively. Thus, both systems demonstrated an acceptable degree of agreement and could be useful in analyzing players’ acceleration demands in professional soccer. However, caution is required when interpreting the results and a comparison with a gold standard is required in order to validate both systems.

## Introduction

The quantification of the demands of competition enables us to improve the individual and team specificity of training load. In recent years, many studies have compared physical demands in professional soccer by analyzing the distances covered by the players at different speeds^1,2^. However, assessing soccer performance only with these variables may underestimate a player’s true workload during a soccer match, due to a lack of consideration of the physiological and mechanical data associated with acceleration and deceleration^3^. The capacity of the muscles to produce acceleration/decelerations and the orientation of ground reaction forces during accelerations and decelerations may determine the appearance of mechanical fatigue^4^, with important implications in soccer performance^5^ and injury risks^6,7^. Understanding the importance of the mechanical load produced by accelerations and decelerations can be fundamental to monitoring activity in soccer. In this regard, the proposal of this study was to quantify accelerations and decelerations in professional soccer matches using two systems.

The challenge of obtaining data on accelerations and decelerations can be addressed using three main technological approaches: (1) Global Positioning System (GPS), (2) Local Positioning System, and (3) semi-automatic multiple-camera Video Tracking Systems (VTS)^8^. GPS is a valid system to measure external load in soccer, which is the psychophysiological response of players to effort, via accelerations, both in training sessions and in matches^9^. However, GPS lacks the accuracy required in measuring explosive accelerations and newer GPS models allow greater analytical filtering^10^, which may be because the commonly used threshold cut-off of 0.6 m/s^2^ results in low acceleration counts^11^. VTS are used to analyze load variables, such as distance traveled and speeds reached^12^. There has been limited research into the use of Video Tracking Systems to record accelerations in soccer, but some evidence supports the validity of using VTS to record accelerations^1,13^.

Various studies have compared accelerometry data from GPS and VTS in training. For instance, Randers et al.^14^ showed significant differences between systems, where GPS tended to provide lower​​ values than VTS in high-acceleration bouts, while this trend was reversed in low acceleration situations. These conclusions are supported by the findings of other pieces of research comparing PROZONE and GPS devices^15,16^. Finally, Linke and Lames^17^ found that neither GPS nor VTS provide valid data for measuring high intensity activity. On this point, it seems necessary to continue investigating the relationship between two devices to quantify the external load on professional soccer players with the aim of achieving a better understanding of the demands of the game. Optical tracking systems and GPS devices could allow us to quantify acceleration and deceleration when assessing external load as it could indicate the mechanical load at the intramuscular and eccentric level^18^. In this sense, not only the number of accelerations or decelerations show the external load caused by these efforts; other parameters like the distances covered with certain acceleration or deceleration values allow to describe more precisely a profile of acceleration/deceleration efforts^18^ or to calculate the high metabolic load distance, a very commonly used variable in understanding the behavior of mechanical variables^19^.

This study was developed in real soccer competition, in contrast to the majority of previous studies that have been developed in non-ecological environments that merely simulate competition conditions^20^. Specifically, the current research is from competition in elite professional players. Moreover, it is from official matches and not from training sessions. Several authors have highlighted the need to quantify these aspects in ecological conditions. In a previous study, it has been suggested that very congested schedules can make decrease the accelerations per minute in elite youth soccer players^21^, supporting the results reported by Akenhead et al.^3^ in professional soccer matches. Also, it has been demonstrated that variations in tactical demands, opponent’s strength or playing style may also influence the results reported^21^. It implies that taking into account the relationship between physical and tactical actions allows a better understanding of the practical applications derived. A combination of VTS and GPS also enables us to include tactical analysis so to understand the accelerations and decelerations profiles.

Therefore, the study set out to evaluate the degree of agreement between two devices, the MEDIACOACH and WIMU PRO, in the number of accelerations and decelerations and the distances covered in each. As a secondary outcome, we also developed mathematical functions that could allow us to exchange data between these models.

## Materials and methods

### Subjects

This study included all 26 professional male soccer players from FC Barcelona B team (mean ± SD age 20.38 ± 2.03 years; height 180.00 ± 7.47 cm; weight 73.81 ± 5.65 kg) (except goalkeepers, due to the specific demands of this position). We recorded the data of the players over the course of all the matches of the 2017/18 Spanish 2nd division (*n* = 42), and a total of 759 measurements were captured by MEDIACOACH^22^ and WIMU PRO^23,24^ at the same time.

### Recordings and procedure

To synchronize both systems on all the players in the team (including substituted players), we used a reconfiguration technique described by Carling^25^. Specifically, before fitting players with the WIMU PRO unit, the device was calibrated and synchronized according to the manufacturer’s recommendations: (1) we turned on the device, (2) waited for ~ 30 s, (3) and once the operating system had initialized, we pressed the record button to start recording, and (4) placed the devices on the player.

In addition, to allow the comparison of data between both systems and to avoid valuation errors, samples were recorded from each half of the matches separately. Due to soccer characteristics, many players remove or change their shirts at half-time and there are signal losses that make synchronization between the two devices impossible during this period of time. More specifically, the datasets from the GPS were adjusted with regard to the VID system (i.e. adjusting the GPS recordings according to the VID registers, always at the beginning of each half). Moreover, data from some matches were deleted because an intermittent signal loss was detected when the raw data were downloaded from the GPS device or the optical tracking camera. Considering first and second halves as separate events, a total of 91 measurements were excluded (13.4%), resulting in a total of 679 measurements analyzed (*n* = 347 first half; and *n* = 332 s half). The matches pertaining to the 29th and 39th rounds were eliminated since these errors of measurement were found in the GPS devices (i.e. outlier data in some players), and the 5th and 10th rounds were eliminated due to visual technical problems detected in the VID.

The study received ethical approval from the second author’s university; Vice-Rectorate of Research, Transfer and Innovation-Delegation of the Bioethics and Biosafety Commission (Protocol number: 153/2017) of University of Extremadura, Spain. Players received verbal and written information regarding the nature of their voluntary participation in the study and informed consent was obtained from all of them. In addition, all participants were treated according to the American Psychological Association ethical guidelines regarding consent, confidentiality, and anonymity.

### Instruments

#### MEDIACOACH

The MEDIACOACH system (Mediapro, Barcelona, Spain) consists of a series of super 4 K-High Dynamic Range cameras based on a positioning system (Tracab-ChyronHego Video Tracking System) that record from several angles and analyze X and Y positions for each player, thus providing real-time three-dimensional tracking. It has been used to quantify soccer demands in previous studies^26–28^. This device is also based on the correction of the semi-automatic VTS (the manual part of the process). This adjustment is made by overlaying the X-coordinate provided automatically by the system for each player onto the actual video image of the match. MEDIACOACH detects and corrects situations in which the positioning coordinates are erroneous because they do not correspond to the position of the individual player in question.

If position datum is completely noise-free, these performance metrics can be computed directly: distance can be recorded frame by frame and the changes in numerical position provide the acceleration values. In the present study, the position data generally contained noise, so this direct approach overestimates distance and speed such that the data need to be filtered to attenuate the effect of noise on the results. To achieve this, we calculated acceleration as the derivative of speed, and applied a smoothing speed signal with a central moving average (rolling average) of two values. After obtaining the acceleration curve, we applied smoothing with a central moving average of 25 values. The MEDIACOACH signal during data collection was 25 Hz (25 frames per second, or 1 data point every 40 ms), so values above 3 m/s^2^ were defined as accelerations. If there was more than one value above the threshold in the same acceleration curve, we retained the highest value. The procedure for recording high-intensity decelerations was the same, but the threshold was − 3 m/s^2^.

#### WIMU PRO

The WIMU PRO device (Realtrack Systems, Almería, Spain) is comprised of different sensors, including four accelerometers, three gyroscopes, a magnetometer, a global navigation satellite system chip (GNSS; *M* = 8.96; *SD* = 1.56) and a UWB chip. It also has a microprocessor, 8 GB of flash memory, a high-speed USB interface to record, store, and share data for further analysis, and an internal battery with four hours of autonomy. It weighs 70 g and measures 81 × 45 × 16 mm. It has been used to quantify soccer demands in previous studies^29,30^. WIMU PRO uses two devices to determine location, and these can be used simultaneously to record the player’s position. In our study (i.e. outdoor conditions), the GNSS determines the position (coordinates) in relation to the time of emission and reception of the signal by radiofrequency on a bandwidth above 500 MHz. The GNSS sampling frequency was 10 Hz. Location technology^23^ and accelerometers^24^ allow us to obtain the distance covered and speed directly, while the other variables analyzed are manually calculated or obtained from other variables. We smoothed the speed and acceleration data using a central moving average of six and four values, respectively. The GPS signal during data collection was 10 Hz (10 frames per second, or 1 data point every 100 ms), so values higher than 3 m/s^2^ (for 100 ms) were considered to be acceleration. Again, if the acceleration curve had more than one value above the threshold, we retained the highest value. Finally, the process of recording high-intensity decelerations was the same used for MEDIACOACH. Also, vests were used, designed specifically to hold the devices, located on the player’s upper torso, and anatomically adjusted to each player, as previously described^31^.

### Measured variables

We collected data on the number of accelerations and decelerations for each player during each match, and the distances they covered. We collected data for all player positions, and for each of the following ranges^32^ we recorded the number of accelerations and decelerations and the distance covered in meters (m):

- Acceleration from 0 to 1 m/s^2^ and deceleration from 0 to − 1 m/s^2^.
- Acceleration from 1 to 2 m/s^2^ and deceleration from − 1 to − 2 m/s^2^.
- Acceleration from 2 to 3 m/s^2^ and deceleration from − 2 to − 3 m/s^2^.
- Acceleration from > 3 m/s^2^ and deceleration from > − 3 m/s^2^.

### Statistical analysis

Data were analyzed using the SPSS Statistics 23.0 software^33^, R-Studio^34^ and Microsoft Excel with a customized spreadsheet from Hopkins^35^. Firstly, the descriptive variables were expressed as the mean and standard deviation (see Table 1). Subsequently, using the Bland and Altman procedure^36^, MEDIACOACH and WIMU PRO statistics were calculated to detect systematic bias ± random error in the sample, and Standard Error of Measurement (SEM) = standard deviation of the sample/√ (sample size) and 95% CLs = Mean ± (SEM × 1.96). Moreover, in order to determine the proportional bias between the methods, we fit a linear regression of the average of the data collection from the two devices with regard to the differences between means, testing β and Cohen’s *f*^2^ method of effect size (see Table 2)^37^.

However, as Hopkins has pointed out^38^, this method could be sensitive to small errors and sample size. Thus, according to Hopkins’ recommendation, a linear regression analysis to compare the error and differences between the systems was conducted to test the validity between the systems. Consequently, we calculated the standardized mean bias (SMB) and the typical error of the estimate (TEE). We rated the SMB as *trivial* (< 0.19), *small* (0.2–0.59), *medium* (0.6–1.19), or *large* (1.2–1.99). We also rated the TEE as *trivial* (< 0.1), *small* (0.1–0.29), *moderate* (0.3–0.59), or *large* (> 0.59). We assessed the level of agreement between the measured criteria by calculating the mean bias^33^ based on 95% CLs (see Tables 3, 4).

In addition, we analyzed the reliability between the WIMU PRO and MEDIACOACH data using the intraclass correlation coefficient (ICC; 1). Values below 0.5 are indicative of *poor* reliability, values between 0.5 and 0.75 indicate *moderate* reliability, values between 0.75 and 0.9 indicate *good* reliability and values above 0.9 indicate *excellent* reliability^39^. We also calculated the percentage least-square means difference (WIMU PRO-MEDIACOACH) and qualitative magnitude-based inference^38^. The magnitude of changes was interpreted as follows: < 0.20 *trivial*, 0.20–0.59 *small*, 0.60–1.19 *moderate*, 1.20–1.99 *large*, 2.0–3.9 *very large*, > 4.0 *extra-large*^25^. The typical error percent coefficient of variation (CV) and the Smallest Worthwhile Change (SWC) for each variable were also estimated. The CV is considered to be the noise of the signal and the SWC the change due to the signal. The recommendation is for the CV to be lower than 10%, although this depends on the context and the data^38^. It is recommended that the SWC be greater than the CV^38^ (see Tables 3, 4).

Finally, a function that would allow us to interchange the data obtained by the two systems was generated. More specifically, a linear regression analysis was conducted estimating the WIMU PRO value that would be expected when we input data from MEDIACOACH for each variable: Y (WIMU PRO data) = (slope × X (MEDIACOACH data)) + intercept (residual errors), where Y is the estimated WIMU PRO value and X is the MEDIACOACH value for a given variable. The intercept represented residual errors in meters (distance variables) or number of accelerations and decelerations (variables). This approach was performed by randomly dividing the set of observation values into k non-overlapping folds of approximately equal size (k = 10). The k-folds validation was carried out in the caret package^40^ which allowed for the comparison of numerous multivariate calibration models under a unified framework. Data pre-processing, parameter tuning, cross validation and model performance evaluation can be performed in this package. The ability of the fitted model to predict the actual observed values was evaluated by the coefficients of determination (R^2^), root mean squared error (RMSE) and mean absolute error (MAE)^41^ The RMSE and MAE error criteria were used to measure the error of the predictions in relation to observed values. MAE is less sensitive to outliers than RMSE. Besides, the R^2^ index was calculated to evaluate the correlation between the predicted and observed values (see Table 5).

## Results

**Table 1:** Descriptive statistics comparing the data obtained by Mediacoach and Wimu systems.

	Number of Acc 0 to 1 m/s^2^	Number of Acc 1 to 2 m/s^2^	Number of Acc 2 to 3 m/s^2^	Number of Acc > 3 m/s^2^	Number of Dec 0 to -1 m/s^2^	Number of Dec -1 to -2 m/s^2^	Number of Dec -2 to -3 m/s^2^	Number of Dec > -3 m/s^2^
Mediacoach^®^	857.6 ± 243.4	147.7 ± 41.4	63.7 ± 18.9	31.7 ± 2.31	868.6 ± 243.5	136.6 ± 69.7	55.2 ± 17.7	36.4 ± 12.1
Wimu Pro™	1041.0 ± 272.8	159.8 ± 45.7	63.6 ± 18.9	29.9 ± 1.98	1033.9 ± 269.7	167.6 ± 70.6	57.9 ± 17.8	34.6 ± 11.7

	Distance Acc 0 to 1 m/s^2^	Distance Acc 1 to 2 m/s^2^	Distance Acc 2 to 3 m/s^2^	Distance Acc > 3 m/s^2^	Distance Dec 0 to -1 m/s^2^	Distance Dec -1 to -2 m/s^2^	Distance Dec -2 to -3 m/s^2^	Distance Dec > -3 m/s^2^
Mediacoach^®^	1052.7 ± 262.4	877 ± 253.5	512 ± 143.3	286.7 ± 98.2	1087.6 ± 273.4	612.9 ± 174.5	268.2 ± 89.0	167.1 ± 63.2
Wimu Pro™	1078.8 ± 269.2	858.5 ± 249.6	498.9 ± 146.9	262.2 ± 95.1	1016.5 ± 253.2	601 ± 170.2	266.4 ± 84.8	173.5 ± 62.9

*Acc*. accelerations, *Dec*. decelerations.

Table 1 summarizes the descriptive statistics comparing the data obtained by the two systems. Regarding the number of accelerations and decelerations recorded at different intensities, WIMU PRO recorded more accelerations and decelerations at low intensities, whereas MEDIACOACH recorded a slightly higher number of accelerations and decelerations at high intensities. With regard to the distances covered at different acceleration and deceleration levels, WIMU PRO recorded a slightly greater distance in accelerations between 0 and 1 m/s^2^ and in decelerations higher than − 3 m/s^2^, whereas MEDIACOACH recorded slightly greater distances traveled in accelerations and decelerations in the remaining ranges.

**Table 2:** Bland and Altman 95% confidence limits of the number and the distance covered in different accelerations and decelerations sections.

Variables	Bias	Estimate (sd*1.96)	Lower CL	Upper CL	*β*	*f*^*2*^
**Number of acceleration–deceleration**						
Acceleration 0/1 m/s^2^	− 181.31	167.06	− 348.37	− 14.25	− 0.34*	0.12
Acceleration 1/2 m/s^2^	− 12.08	40.06	52.15	27.98	− 0.21*	0.04
Acceleration 2/3 m/s^2^	0.14	17.45	− 17.31	17.59	0.00	0.00
Acceleration > 3 m/s^2^	1.79	10.14	− 8.36	11.93	− 0.02	0.00
Deceleration 0/− 1 m/s^2^	− 165.31	161.74	− 327.06	− 3.57	− 0.32*	0.11
Deceleration − 1/− 2 m/s^2^	− 31.17	46.05	− 77.23	14.88	− 0.30*	0.09
Deceleration − 2/− 3 m/s^2^	− 2.66	16.92	− 19.58	14.27	− 0.01	0.00
Deceleration > − 3 m/s^2^	1.81	11.63	− 9.82	13.44	0.07	0.00
**Distance in acceleration–deceleration**						
Acceleration 0/1 m/s^2^	− 25.83	119.08	− 144.91	93.25	− 0.10	0.01
Acceleration 1/2 m/s^2^	18.44	138.71	− 157.15	120.26	0.05	0.00
Acceleration 2/3 m/s^2^	− 13.09	128.43	− 141.52	114.34	− 0.03	0.00
Acceleration > 3 m/s^2^	− 24.50	93.43	− 117.94	68.93	− 0.06	0.00
Deceleration 0/− 1 m/s^2^	71.15	134.04	− 205.19	62.89	0.29*	0.08
Deceleration − 1/− 2 m/s^2^	11.98	111.57	123.55	99.59	0.07	0.00
Deceleration − 2/− 3 m/s^2^	− 1.82	86.64	− 88.47	84.82	− 0.09	0.01
Deceleration > − 3 m/s^2^	− 5.49	56.82	− 50.33	63.30	0.01	0.00

In Table 2, according to Bland and Altman^36^, the linear regression presents a proportional (i.e. not constant) bias in some variables. More specifically, negative biases were found in the number of low-intensity acceleration (0/1 m/s^2^, β = − 0.34, *p* < 0.05; and 1/2 m/s^2^, β = − 0.21, *p* < 0.05), and deceleration sections (0/− 1 m/s^2^, β = − 0.32, *p* < 0.05; and − 1/− 2 m/s^2^, β = − 0.30, *p* < 0.05), whereas negative biases were found in 0/1 m/s^2^ in distance covered and in all high-intensity acceleration and deceleration sections. This indicates that the measurement error decreases as the recorded score increases in most variables. Also, the effect size calculated for the *f*^2^ value ranged between 0.00 and 0.12 in the number of acceleration and deceleration sections and between 0.00 and 0.08 for the distance covered in each one, all considered small^37^.

**Table 3:** Comparison of each variable in the number of acceleration/deceleration in different sections analyzed by Mediacoach and Wimu data, including standardized mean bias, typical error of estimate (TEE), intraclass correlation coefficient (ICC), % change mean, % smallest effect and % coefficient of variation (CV), of all with 95% confidence limits.

	Number of Acc. 0 to 1 m/s^2^	Number of Acc. 1 to 2 m/s^2^	Number of Acc. 2 to 3 m/s^2^	Number of Acc. > 3 m/s^2^	Number of Dec. 0 to -1 m/s^2^	Number of Dec. − 1 to − 2 m/s^2^	Number of Dec. − 2 to − 3 m/s^2^	Number of Dec. > − 3 m/s^2^
**Agreement analysis**								
Standardised mean bias	− 0.66	− 0.26	0.01	0.17	− 0.61	− 0.68	− 0.15	0.15
95% CLs	[− 0.68 to − 0.64]	[− 0.29 to − 0.23]	[− 0.02 to 0.04]	[0.14 to 0.21]	[− 0.63 to − 0.59]	[− 0.72 to − 0.65]	[− 0.18 to 0.12]	[0.12 to 0.18]
	*Moderate*	*Small*	*Trivial*	*Trivial*	*Moderate*	*Moderate*	*Trivial*	*Trivial*
Standardised TEE	0.32	0.50	0.51	0.57	0.32	0.36	0.54	0.55
95% CLs	[0.31 to 0.35]	[0.47 to 0.54]	[0.48 to 0.58]	[0.53 to 0.61]	[0.30 to 0.34]	[0.33 to 0.38]	[0.51 to 0.58]	[0.52 to 0.59]
	*Moderate*	*Moderate*	*Moderate*	*Moderate*	*Moderate*	*Moderate*	*Moderate*	*Moderate*
ICC	0.98	0.92	0.92	0.90	0.98	0.92	0.92	0.90
95% CLs	[0.97 to 0.98]	[0.91 to 0.93]	[0.91 to 0.93]	[0.89 to 0.91]	[0.97 to 0.98]	[0.91 to 0.93]	[0.91 to 0.93]	[0.89 to 0.91]
	*Excellent*	*Excellent*	*Excellent*	*Good*	*Excellent*	*Excellent*	*Excellent*	*Good*
% Change means	− 12.8 *Moderate*	− 7.1 *Small*	0.5 *Trivial*	6.8 *Trivial*	− 11.3 *Moderate*	− 13.2 *Moderate*	− 4.8 *Trivial*	4.1 *Trivial*
% Smallest effect	8.1	7.6	8.2	8.3	8.0	7.6	8.2	8.3
% Typical error (CV)	6.1	7.3	9.6	13.4	6.0	7.4	9.8	13.7

*Acc*.  acceleration, *Dec*. deceleration, *CLs* confidence limits.

Comparing the number of accelerations^35,38,39^ detected by the two instruments (Table 3), we found *trivial* and *small* differences in SMB, except for the differences recorded in the number of accelerations from 0 to 1 m/s^2^ and decelerations below ± 2 m/s^2^ (i.e. *moderate*). Regarding the standardized TEE, all variables presented *moderate* scores. The ICC presented good agreement between both systems, with values above or close to 0.90, classified as *good* (i.e. in the number of accelerations and decelerations at more than 3 m/s^2^), and as *excellent* (i.e. in the other acceleration and deceleration ranges). The magnitude of change in means (%) was *moderate* in low-intensity accelerations and decelerations and *small* to *trivial* in high-intensity accelerations and decelerations. The SWC (%) obtained scores ranging between 7.6 and 8.3%. Finally, the % of CV for all variables was between 6 and 13.7%.

**Table 4:** Comparison of each variable in distances covered in different acceleration/deceleration sections analyzed by Mediacoach and WIMU data, including standardized mean bias, typical error of estimate (TEE), intraclass correlation coefficient (ICC), % change mean, % smallest effect and % coefficient of variation (CV), all with 95% confidence limits.

	Distance Acc. 0 to 1 m/s^2^	Distance Acc. 1 to 2 m/s^2^	Distance Acc. 2 to 3 m/s^2^	Distance Acc. > 3 m/s^2^	Distance Dec. 0 to − 1 m/s^2^	Distance Dec. − 1 to − 2 m/s^2^	Distance Dec. − 2 to − 3 m/s^2^	Distance Dec. > − 3 m/s^2^
**Agreement analysis**								
Standardised mean bias	− 0.10	0.07	0.09	0.25	0.28	0.07	0.02	− 0.10
95% CLs	[− 0.11 to − 0.08]	[0.05 to 0.09]	[0.07 to 0.12]	[0.22 to 0.28]	[0.26 to 0.30]	[0.05 to 0.09]	[0.00 to 0.05]	[− 0.13 to − 0.07]
	*Trivial*	*Trivial*	*Trivial*	*Trivial*	*Small*	*Trivial*	*Trivial*	*Trivial*
Standardised TEE	0.23	0.27	0.48	0.54	0.25	0.24	0.53	0.50
95% CLs	[0.22 to 0.25]	[0.25 to 0.29]	[0.45 to 0.51]	[0.51 to 0.58]	[0.24 to 0.27]	[0.22 to 0.27]	[0.50 to 0.56]	[0.47 to 0.53]
	*Small*	*Small*	*Moderate*	*Moderate*	*Small*	*Small*	*Moderate*	*Moderate*
ICC	0.99	0.97	0.94	0.96	0.98	0.96	0.93	0.95
95% CLs	[0.98 to 1.00]	[0.96 to .98]	[0.93 to 0.95]	[0.95 to 0.97]	[0.97 to 0.99]	[0.95 to .97]	[0.92 to 0.94]	[0.94 to 0.96]
	*Excellent*	*Excellent*	*Excellent*	*Excellent*	*Excellent*	*Excellent*	*Excellent*	*Excellent*
% Change means	− 2.4 *Trivial*	2.1 *Trivial*	− 2.8 *Trivial*	− 7.0 *Small*	6.1 *Small*	2.0 *Trivial*	− 0.2 *Trivial*	− 4.1 *Trivial*
% Smallest effect	8.1	7.6	8.2	8.3	7.6	7.4	8.6	8.3
% Typical error (CV)	4.3	6.3	9.3	13.5	4.7	6.4	9.1	14.3

*Acc*. acceleration, *Dec*. deceleration, *CLs* confidence limits.

Table 4 describes the data for each variable in distances covered in different acceleration/deceleration zones analyzed by MEDIACOACH and WIMU PRO^36,38,39^. We observed that the mean distances covered in accelerations or decelerations showed only a *trivial* difference between the two systems, with the exception of a *small* difference for decelerations of 0 to − 1 m/s^2^. As for the standardized TEE, all variables for distances covered in accelerations and decelerations varied between *small* (in higher acceleration and deceleration intervals) and *moderate* (in smaller acceleration and deceleration intervals). The ICC presented good agreement between both systems, with values above 0.90 for all variables (i.e. *excellent*). The magnitude of change in mean (%) ranged from small to *trivial* or *small* in all variables. The SWC (%) oscillated between 7.4 and 8.6%. Finally, a CV (%) of between 4.3% and 14.3% was obtained for all variables.

As we pointed out in the statistical analysis section, a function that would allow us to interchange the data obtained by the two systems was generated:

Y (WIMU PRO data) = (slope × X (MEDIACOACH data)) + intercept (residual errors), where Y is the estimated WIMU PRO value and X is the MEDIACOACH value for a given variable. The intercept represents the residual errors of the number of accelerations and decelerations and distances covered in m/s^2^. This was then compared to randomized sample to examine whether the equation fit correctly and to determine whether the application of the values to MEDIACOACH was valid compared to the WIMU PRO values.

**Table 5:** The regression equations and cross-validation in distance covered and number of accelerations and decelerations in different sections.

Number of acceleration and deceleration	R^2^	RMSE	MAE	Distance covered	R^2^	RMSE	MAE
*Acceleration 0/1 m/s*^*2*^: Y = 1.07x + 117.2	0.92 (0.89–0.93)	70.01 (64.33–76.88)	63.34 (58.85–69.11)	*Acceleration 0/1 m/s*^*2*^: Y = 0.9x + 29.02	0.95 (0.93–0.96)	.60.61 (56.01–63.88)	45.71 (43.61–49.29)
*Acceleration 1/2 m/s*^*2*^: Y = 0.99x + 14.95	0.88 (0.84–0.92)	20.71 (18.72–22.08)	15.82 (14.38–17.34)	*Acceleration 1/2 m/s*^*2*^: Y = 0.95x + 29.33	0.93 (0.91–0.94)	68.15 (66.16 (70.15)	52.70 (50.49–54–41)
*Acceleration 2/3 m/s*^*2*^: Y = 0.88x + 7.75	*0.89 (0.85–0.95)*	8.51 (7.87–9.01)	6.51 (6.18–7.09)	*Acceleration 2/3 m/s*^*2*^: Y = 0.89x + 43.83	0.91 (0.85–0.94)	61.92 (58.52–64.17)	50.47 (47.52–52.35)
*Acceleration >3 m/s*^*2*^: Y = 0.85x + 3.97	0.85 (0.76–0.93)	5.65 (5.26–6.36)	4.35 (3.98–4.71)	*Acceleration >3 m/s*^*2*^: Y = 0.84x + 21.26	0.0.85 (0.80–0.90)	45.55 (42.51–47.32)	35.25 (33.37–37.38)
*Deceleration 0/− 1 m/s*^*2*^: Y = 1.01x + 164.48	0.91 (0.87–0.93	69.78 (62.97–75.31)	64.04 (58.94–70.10)	*Deceleration 0/− 1 m/s*^*2*^: Y = .90x + 42.296	0.94 (0.92–96)	59.49 (56.12–63.18)	47.62 (42.89–48.87)
*Deceleration − 1/− 2 m/s*^*2*^: Y = 0.99x + 32.29	0.85 (0.80–0.90)	23.30 (20.59–26.18)	18.06 (16.56–20.03)	*Deceleration − 1/− 2 m/s*^*2*^: Y = 0.91x + 40.10	0.91 (0.93–0.89)	53.94 (49.92– 55.97)	41.83 (39.05–43.15)
*Deceleration − 2/− 3 m/s*^*2*^: Y = 0.88x + 9.47	0.87 (0.82–90)	8.59 (8.13–9.21)	6.71 (6.42–7.24)	*Deceleration − 2/− 3 m/s*^*2*^: Y = 0.89x + 9.93	0.87 (0.82–0.90)	8.51 (8.28–8.88)	6.58 (6.38–6.77)
*Deceleration >− 3 m/s*^*2*^: Y = 0.90x + 5.36	0.87 (0.81–0.90)	5.65 (5.10–6.01)	4.42 (4.01–4.82)	*Deceleration >− 3 m/s*^*2*^: Y = 0.88x + 28.66	0.89 (0.83–0.94)	28.65 (27.22–29.22)	23.15 (21.91–23.73)

*R*^*2*^ R-square, *RMSE* root mean squared error, *MAE* mean absolute error.

Finally, Table 5 also shows the R^2^, RMSE and MAE of the number and distances covered in different acceleration and decelerations sections. The proposed models showed an adequate fit, with R^2^ higher than 0.85 in all variables, and values of RMSE and MAE lower than 10% of error.

## Discussion

The aim of this study was to compare the accelerations and decelerations values obtained during official competitions recorded in a team by MEDIACOACH and WIMU PRO, examining the degree of agreement between these systems in the number of accelerations and decelerations and the distances covered in meters. The results showed an adequate degree of concordance between WIMU PRO and MEDIACOACH for all variables measured, although caution is still required when interpreting accelerations/decelerations > 3 m/s^2^.

First, taking the variables analyzed in this study into account, to our knowledge this is the first work that analyzes accelerations in the ranges proposed here. García-Unanue et al.^42^ defined, using GPS, the number of high-intensity accelerations as anything above 2.75 m/s^2^, and obtained an average of ~ 30 high-intensity accelerations per game. Other authors^43^ also found 597.43 ± 31.68 (numbers, in defenders) and 610.75 ± 32.06 (numbers, in midfielders) accelerations over 1.5 m/s^2^ in a Bundesliga analysis, and these results are similar to those obtained in this study with WIMU PRO and MEDIACOACH.

Regarding the results obtained comparing both systems in the number of accelerations and decelerations in the different intensities sections, WIMU PRO provides higher values than MEDIACOACH at low intensities (< 2 m/s^2^). In contrast, MEDIACOACH recorded slightly higher values than WIMU PRO in high-intensity accelerations and decelerations. This difference has also been reported by other authors^15,16^ and could be because the systems differ in considering when the acceleration starts. While MEDIACOACH only records movements (and therefore accelerations) when there has been a minimum displacement of one meter, WIMU PRO records an acceleration when there is a specific difference in the speed derivative^43^. For this reason, WIMU PRO could overestimate acceleration values ​​in the medium and low range^44^.

Regarding the level of agreement, we found an adequate level of agreement in SMB and in the number of accelerations from 0 to 1 m/s^2^ and decelerations below ± 2 m/s^2^. More agreement problems were showed between instruments, but it is over the minimum statistics threshold. Again, the overestimation of WIMU PRO in low intensities is noteworthy. These results are in line with previous research^45^ comparing different GPS devices. This study showed that the change of mean was greater at low intensities compared to high intensities in the number of accelerations and decelerations recorded. Thus, while we can understand that the change of means above 10% could be high for the agreement between the instruments, this could easily be remedied by modifying the procedure to account for the start of an acceleration. On the other hand, GPS presented poor accuracy at high-intensity acceleration/deceleration^46^, which can generate more noise and difficulty in interpreting the signal.

Regarding the distances covered at different ranges of acceleration and deceleration, WIMU PRO recorded a slightly greater distance in accelerations of 0–1 m/s^2^, and in decelerations above − 3 m/s^2^, whereas MEDIACOACH slightly overestimated the distance for the other accelerations and decelerations. For all distances covered in accelerations and decelerations, there was *agreement* between the means showed by MEDIACOACH and WIMU PRO, except in decelerations of 0 to − 1 m/s^2^, which showed a *small difference*. Our results are consistent with previous findings^44^. In general, these previous results presented a better agreement between the systems in distance covered than in number, in all acceleration and deceleration sections. Most research and applications focus on analyzing distances covered in meters instead of the number of accelerations and decelerations recorded in official matches, which is why the degree of agreement between both systems facilitates the interpretation from this perspective.

Note that this is the first study that compares accelerations/decelerations from a VTS to those from a GPS system using real tracking data in official competitions. Most previous studies have merely simulated competition conditions^20^. Indeed, we would like to highlight this point. Although this research demonstrates good agreement between both instruments, the results should also be interpreted with caution, especially at high-acceleration intensities. These findings could be useful for researchers and practitioners to be able to make progress in terms of knowledge and application with data measured from an ecological perspective.

### Limitations and practical applications

This study has some limitations that should be considered in order to correctly interpret the results. First, it is difficult to validate accelerations and decelerations^15^. Although the reliability and accuracy of GPS have improved, they still have limitations, particularly in the context of changes of direction or curvilinear movements in high intensity activity^15,47^. The present investigation has used different methodological approaches at the statistical level, demonstrating that it is necessary to be cautious with the greater changes in accelerations/decelerations values.

Secondly, another important question is that 91 of the 679 measurements had to be excluded. This involved the loss of 13.4% of the total data. Hoppe et al.^48^ lost 10% and 20% of the measurements with 10 Hz GPS and 18 Hz GPS, respectively. In this regard, Linke et al.^49^ recorded 6.3% for GPS and 4.6% for VID measurement errors. In this study, errors were found for GPS signals in the 29th and 39th rounds. Siegle et al.^50^ explained that positioning systems can be negatively influenced by weather and stadium characteristics (e.g. high stands). Other problems, such as indoor stadiums, were related to GPS^24^, but there were no fully-covered stadiums in the competition analyzed. Moreover, problems with VID in the study were related to visual technical problems in the 5th and 10th rounds. Thus, teams that only use one system regularly should bear in mind the characteristics of any given stadium.

On the other hand, these results could facilitate several interesting applications for researchers and practitioners. Firstly, and as is already stated elsewhere^15^, all systems have their advantages and disadvantages, and the ideal tracking system has yet to be developed. Interestingly, here we developed a predictive equation that allows us to exchange data between the two systems used. To adequately evaluate a player’s overall movement load, and to integrate data from different systems accordingly, practitioners should use calibration equations. In our study, we validated the equations that allowed us to estimate the number and distances covered in different acceleration and deceleration sections provided by WIMU PRO with data recorded using MEDIACOACH, and vice versa (i.e. cross-validation). The interchangeability of these types of systems is important for practitioners in professional clubs who often use two different systems during the week.

Secondly, although this study is a typical case in a professional football club, many other teams currently have access to MEDIACOACH and/or use WIMU PRO to quantify the external load of their teams during training sessions and matches. More specifically, these two devices have been currently used by several professional soccer clubs and national teams in countries like Spain, Mexico or Russia. It is important that coaches use and understand this information in the context of the particular stage of the season, as well as the characteristics of the team. These data could be essential to the daily work of coaching and medical staff and other professionals involved in looking after athletes’ health. In addition, this indirect calculation system (i.e. Tracab Optical Tracking-CHYRONHEGO) related to acceleration and deceleration variables could also be used in the 300 stadia currently part of the English Premier League, German Bundesliga and Spanish LaLiga, among other professional soccer leagues (i.e. around 4500 professional matches a year). Moreover, WIMU PRO is used by several soccer teams in different countries (e.g., Spain, Mexico or Russia).

Additionally, due to the problems presented by GPS in certain conditions, and the fact that soccer players would rather be measured by non-portable instruments^39^, the results of this investigation could overcome these limitations. Specifically, the issues reported by players with portable instruments have been in competitive situations. MEDIACOACH resolves these problems, however these instruments are prone to measurement problems in certain conditions, as mentioned. Since we know which contexts are more difficult for MEDIACOACH measurements, we can recommend to coaches the use of GPS in these cases and the possibility of interchanging the information between systems. Research is currently focusing on comparing related instruments that permit data interchangeability^12,22,45,51^. To this end, FIFA Football Technology Innovation Department is working on the standardization of electronic performance and tracking systems (EPTS), thereby seeking to provide guidance to football stakeholders regarding the use of EPTS in competitive matches^52^.

Thirdly, these results could render it possible to fill the gap encountered by strength and conditioning coaches, reconditioning and performance coaches and medical staff during official competitions, helping them to plan training loads based on the accelerations and decelerations, made during competition with a view to optimizing player performance, reducing risk of injury and return to fitness (through effective monitoring of the effort and fatigue of the players). Specifically, different performance indicators may enable such calculations as the high metabolic load distance. Also, the scientific community could have access to performance data recorded during official matches without the need to simulate situations. Of course, it is never possible to reproduce precisely what happens in a real match. Furthermore, the differences found in this comparison could be associated with the error obtained in other research works that have compared other similar instruments^15,49,53^.

Finally, the equations that allow the exchange of data between the WIMU PRO and MEDIACOACH would allow clubs to incorporate a broader dimension into performance analysis, reducing the loss of information due to technical issues with the instrument and responding to the demands of both player and team, thus facilitating closer monitoring of aspects such as trends in physical demands over time.

## Conclusion

To conclude, this study succeeded in demonstrating more than a minimum threshold of agreement between these two systems (WIMU PRO and MEDIACOACH) used to analyze players’ acceleration demands in professional soccer. However, caution is still required when interpreting accelerations/decelerations > 3 m/s^2^. Although WIMU PRO gave slightly higher acceleration and deceleration values at low intensity compared to MEDIACOACH, both systems showed adequate agreement and were considered as useful instruments for recording players’ load during official matches. The linear regression showed slightly overestimated values in favor of MEDIACOACH, being consistent with the findings of most of the previous related studies^22,27^. Therefore, the agreement shown by these two systems allows the quantification of external load in professional soccer players (i.e. both in training sessions and in matches) to evolve the greater knowledge about the accelerations and decelerations (biomechanics) responses and loads responses.

### Permission statement

The authors declare that the appropriate permission was obtained from MEDIACOACH and WIMU PRO to use these systems in this research and in subsequent publications.
